# Dangerous Popular Cures

**Published:** 1909-10-09

**Authors:** 


### The General Practitioner's Column.

[Contributions to this Column are invited, and if accepted will be paid for.]

### DANGEROUS POPULAR CURES.

It not unfrequently happens that a practitioner finds, when called to see a patient, that he is not so much suffering from any injury or disease, as from the effects of some popular remedy which has been applied. Such remedies occasionally prove dangerous to health and life, and merit a little regard.

Some years ago I pointed out in a contemporary journal * that a spider's web, an old cure for bleeding, is an uncleanly application, and it is generally procured from the most neglected corner in a room, and is consequently laden with dust. The following note illustrates this fact:—

*Cobwebs as "Cure."*—The application of cobwebs to stop bleeding from a wound on the index finger of the left hand, received whilst feeding a chaff-cutter, caused the death from lockjaw of William Philpott, aged sixty-five, of Minster, Sheppey. Tetanus symptoms developed eleven days after the accident. At the inquest yesterday, when a verdict of accidental death was returned, the county coroner said he hoped the case would be a warning to people not to apply cobwebs to wounds.—*Standard*, March 7, 1906.

The earliest reference to this remedy in our language seems to be in a translation of that curious encyclopædic work of the Middle Ages, *De Proprietatibus Rerum*, where we read: "Coppe webbe that is white and clene staunchyth blood." But as I have seen it applied to a cut finger it has been anything but white and clean. There is another reference in Shakespeare's *Midsummer Night's Dream*, where Bottom, the weaver, says to the fairy Cobweb, "I shall desire of you more acquaintance, good master, Cobweb; if I cut my finger I shall make bold with you."

As a styptic, however, it must be acknowledged a spider's web is somewhat effective. In a case of excessive hæmorrhage after the extraction of a tooth a dentist applied a cobweb with most satisfactory results.

Among country people there is a great belief in animal cures, and the reckless way in which they apply the flesh of dead animals to their own bodies cannot be unattended with some risk of septic poisoning. A butcher tells me that persons affected with asthma often ask to be allowed to envelope themselves in the disembowelled carcass of a freshly-killed animal, declaring that they get much benefit from such treatment; and rheumatic invalids beg to have paunches to wrap round their swollen joints. In my first experience of practice in Kent I was puzzled when I was informed by patients that they had put a "flead" on their sores or wounds. A "flead" I now learn is the inside fat of a hog before it is melted and strained and made into lard, and, therefore, is impure. The word is most commonly used in Kent and Sussex. While I was a student at a hospital in London a countryman had his leg amputated on account of the mischief arising from the application of a cow-dung poultice, which was probably infested with the larvæ of flies and beetles. Such a poultice has been highly esteemed in the north, as may be seen from the mention made of it in a dialect poem by a Cumbrian poet, "Ann, git cow-scairn. . . . Nowt maks a pultess better."

There are several popular cures for a black eye, both animal and vegetable, as raw beef, Solomon's seal, and arnica. I have seen ill effects from the use of the last. Very recently a charwoman was chopping firewood when a splinter struck her under the eye. Some tincture of arnica was dropped into an egg-cup containing water, and the solution applied. When I saw her a few days after the accident she looked just as if she was suffering from an acute attack of erysipelas in the face. There was so much swelling around both eyes that she could hardly see out of them, and the inflammation spread down her neck. But there was no rise of temperature or delirium at night, and in about a week she recovered. According to Whitla, arnica externally applied may set up fatal inflammatory action, and at best is an uncertain and often a dangerous remedy, and therefore ought to have been omitted from the new B.P. I know a chemist who refuses to supply the drug to his customers, having seen such bad consequences from its use, and yet he states his wife would always persist in applying the remedy when any of her children got bruises. On the continent the arnica plant has been called leopard's bane, and in some of its properties it resembles wolf's bane or aconite. It is said that no animal but the goat will eat this poisonous plant.

Some alarming revelations have been made lately regarding a powder commonly applied to moist and excoriated parts. A London coroner, Dr. Wynn Westcott, held two inquiries in cases of infants who had died of tetanus, and in each case fuller's earth had been applied to the navel and nates; and he remarked that he had about four such cases every year, and always found that fuller's earth had been used as a dusting powder. Fuller's earth is a soft, friable greenish or yellowish grey kind of clay and contains silica of alumina and oxide of iron. It is found in Kent, Surrey, Bedfordshire, Nottinghamshire, and at Maxton in Scotland. In "fulling" it has been largely employed for extracting oil and grease from cloth, and was so much valued in bygone times that laws were enacted prohibiting its exportation. Its well-known cleansing properties have made it a figure of speech. Thus an old divine in the seventeenth century writes: "The blots of sin will be easily taken out by the soap of sorrow and the fuller's earth of contrition." After being dug up the earth is well dried in ovens, and then thrown into cold water, when it falls to powder, and lastly is purified by washing over. If this process is thoroughly carried out, it is surprising that the earth should not be completely sterilised. But it is often supplied in crude lumps to the poorer classes, who dry and convert them into a powder in their own rough way.

* Chambers, Jan. 19, 1895.

